# A bibliometric analysis on traumatic brain injury in forensic medicine of a half-century (1972–2021)

**DOI:** 10.3389/fneur.2023.913855

**Published:** 2023-02-02

**Authors:** Yufang Wang, Qianqian Chen, Xingxing Dang, Wanqing Lu, Xinran Zhang, He Yan, Shuliang Niu, Xisheng Yan, Jie Yan

**Affiliations:** ^1^Department of Forensic Science, School of Basic Medical Science, Central South University, Changsha, Hunan, China; ^2^School of Basic Medical Science, Xinjiang Medical University, Urumqi, China; ^3^Department of Cardiovascular Medicine, Wuhan Third Hospital, Tongren Hospital of Wuhan University, Wuhan, Hubei, China

**Keywords:** traumatic brain injury, bibliometric analysis, diffuse axonal injury, shaken baby syndrome, concussion

## Abstract

Traumatic brain injury (TBI) is among the most common injuries in forensic medicine, the identification of which is of particular importance in forensic practice. To reveal the circumstances and trends of TBI in the forensic field, we used the Web of Science (WoS) database for comprehensive retrieval. We made a metrological analysis of 1,089 papers in the past 50 years (1972–2021). The United States and Germany have the most forensic research on TBI. Diffuse axonal injury (DAI) has been the focus of attention for many years, and much effort has been devoted to its diagnosis in forensic pathology. Infants and children are the subgroups of most concern, especially in infant and child abuse cases. Research on identifying shaken baby syndrome has received increasing attention in recent years. Overall, our study provides a comprehensive list and analysis of the articles regarding TBI in legal medicine, which may shed light on recognizing the trends and research hotspots in this field.

## 1. Introduction

Traumatic brain injury (TBI) is a medical condition with high morbidity worldwide, with extremely high mortality and disability rates. In children and young adults, TBI is the leading cause of death and disability (1). A TBI-related death usually results from self-harm, traffic accidents, and falls (2). TBI comprises diffuse axonal injury (DAI), contusion, fracture, epidural hematoma, subdural hematoma, and diffuse brain edema according to biomechanical and neuroradiological features, among which DAI is one of the most common types (3). DAI was regarded as one of the essential primary brain injuries of blunt TBI, and its diagnosis has important forensic significance.

In general, TBI does not necessarily result in rapid patient death. However, secondary injuries after TBI can worsen the patient's condition and even cause death (4). Subdural hematomas, familiar clinical entities after TBI, can lead to delayed death. Because the hematoma arises from a vein, it can initially be asymptomatic but cause death within days or months (5). In addition, both DAI and subdural hematomas are typically associated with abused infants. In the absence of visible signs of head impact, such as contusion or laceration, it is usually challenging to correctly identify whether there is a TBI, analyze the relationship between injury and disease, and make a judgment on the cause of death (4). Further understanding of its current status may facilitate the development of revealing death causes in forensic identification, particularly when it comes to abused infants.

Over the past few decades, forensic medicine has published thousands of research articles on TBI, and assessing the scientific literature on the subject is essential for forensic scientists. Bibliometrics is a quantitative analysis method of scientific documents (6, 7), which has been widely applied in the study of areas such as cardiovascular, hematology, neurology, etc. Recently, similar methods of analysis have been performed regarding severe TBI (8) and subarachnoid hemorrhage (6). However, to our knowledge, such studies still lack in the current forensic science area. Herein, we employed the bibliometric method to visually analyze the forensic literature on TBI in the recent 50 years. We wish to provide new insights into the postmortem diagnosis of TBI.

## 2. Materials and methods

### 2.1. Data collection

**Figure 1:**
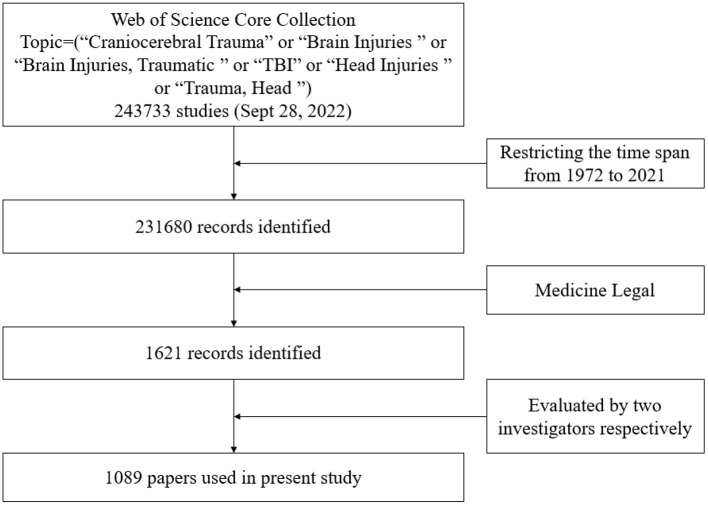
Flow chart of case selection.

We performed a literature search using the Web of Science (WoS) Core Collection Database on September 28, 2022. Search terms and strategy search words used were “Craniocerebral Trauma” or “Brain Injuries” or “Brain Injuries, Traumatic” or “TBI” or “Head Injuries” or “Trauma, Head”. Topic Subject was used as a retrieval field. All categories of publications were considered, and no time restrictions were placed. A total of 243,733 papers were retrieved, of which 231,680 reports were from 1972 to 2021. We limited the publications in the field of TBI to all those indexed under the research category “Medicine Legal” in the WoS database and identified 1,621 papers. Two independent investigators evaluated all documents, focusing on titles and abstracts to verify that the documents were related to TBI. If necessary, the investigators read the full text to decide on inclusion. Finally, 1,089 papers were included and exported from the WoS (Figure 1). The “full Records and citations” were derived as metadata, which was further imported to VOSviewer for analysis. In addition, the “Results Analysis” tool of the WoS was applied to derive relevant data to Excel, presenting the information on publication years, document types, funding agencies, authors, source titles, countries/regions, and institutions.

### 2.2. Analysis

All the information in the retrieved publications, including the titles, authors, journals, abstracts, keywords, and citations, were imported to VOSviewer 1.6.14 for analysis. Additionally, we imported the publication information required to Microsoft Excel 2013 (Microsoft, Redmond, USA) for further data handling. To show the research status of TBI in forensic medicine, we employed the following indicators: the number of published papers per year, including average, median, standard deviation, and quartiles; top 10 authors, countries, institutions, and funding agencies with the most published, top 10 keywords used most frequently, and top 10 papers most cited. The annual growth rate (AGR) and relative growth rate (RGR) (9) of a specific interval were calculated and plotted using Microsoft Excel 2013 from the following formulas: (1) AGRij = (Ni – Nj)/Nj. (2) RGRij = (ln Ni – ln Nj)/(Ni – Nj). Where Ni in Equations (1) and (2) is the final number of documents published in the year, Nj in Equations (1) and (2) is the number of documents published in i – 1 year.

We used VOSviewer 1.6.14 to construct visualization network maps and density maps to show between-country cooperation, the degree to which journals cite each other, and the co-occurrence of keywords and terms. In network visualization maps, the thickness of the line between two items reflected the strength of the relationship based on the number of lines between the two items (10, 11). The journal impact factors (IF) were identified through the 2021 Journal Citation Reports (JCR). The Scimago Graphica (1.0.18) was used to perform collaboration networks between countries. R-Tool Bibliometrix (version 4.0.1) of R-Studio (version 4.2.1) and bibliophily, which provided a Web interface for Bibliometrix, were used to summarize the relationships between keywords, references authors use, and the top authors by a three-fields plot.

## 3. Results

We searched 243,733 documents in 50 years, of which 1,089 were published in forensic journals. Of the 1,089 papers, most were articles (81%) (Supplementary Figure 1), and a majority of them were published in English (94.12%), followed by German (5.14%), French (0.50%), and unspecified language (nasty 0.37%).

### 3.1. Annual output of publications

**Figure 2:**
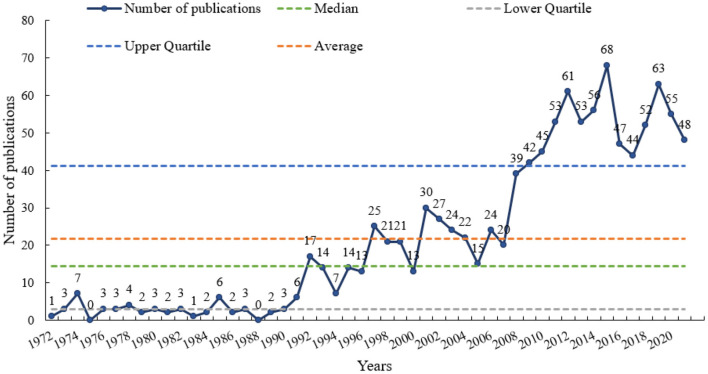
Annual publication trends from 1972 to 2021.

From 1972 to 2021, the number of published papers showed a trend of a wavelike rise. Except for 1972 and 1988, at least one document was published each year. The first year with more than ten papers was 1992. Sixty-eight documents were published in 2015, the most productive year (Supplementary Table 1). There were 21.78 ± 20.75 papers annually, with a median of 14.5 papers, three papers with lower quartile, and 41.25 papers with upper quartile (Figure 2). In the first 20 years (1972–1991), 56 papers (5.14%) were published. One hundred and seventy-five (16.10%), 311 (28.56%), and 547 (50.23%) papers were issued during the decades of 1992–2001, 2002–2011, and 2012–2021, respectively.

### 3.2. Primary countries or regions

**Figure 3:**
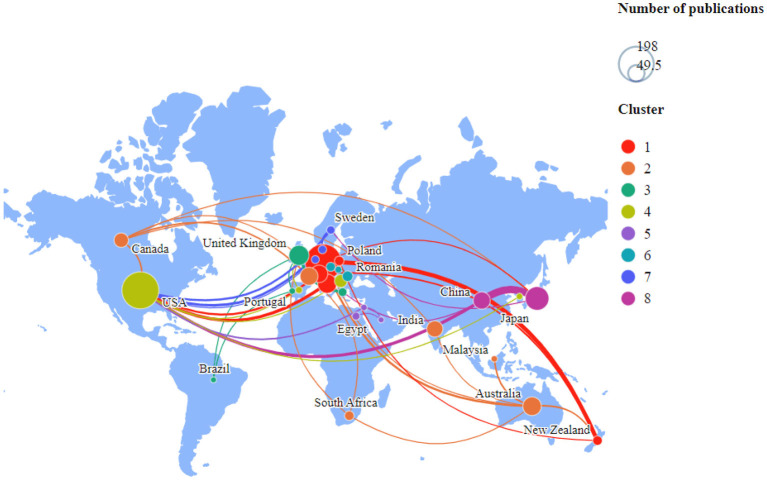
Distribution of countries and regions contributed to research on TBI in legal medicine. The size of the circles indicates the number of publications. The link strength between the circles reflects the frequency of co-occurrence.

As shown in Figure 3, 61 countries have participated in the publications on forensic TBI, among which 21 countries have published more than ten papers. The top three countries are the United States (198, 18.18%), Germany (192, 17.63%), and Japan (81,4.68%). The top ten countries published 804 papers, accounting for 73.83% of the total. According to the list of developed countries published by the United Nations, eight of the ten are developed countries, and only China and India are developing countries (Supplementary Figure 2). We analyzed the cooperation relationship between countries through the ties of co-authors in 32 countries (Figure 3). The visual map of national cooperation identified 66 links and eight clusters: (1) Austria, Germany, Italy, New Zealand, Poland, and Switzerland; (2) Australia, France, Canada, India, South Africa, Malaysia; (3) United Kingdom, Greece, Wales, Portugal, Brazil; (4) USA, Serbia, Spain, South Korea; (5) Egypt, Israel, Saudi Arabia; (6) Romania, Czech Republic, Slovakia; (7) Denmark, Sweden, Netherlands; (8) Japan, China. Japan has the closest cooperation with China (link strength, 13). In addition, Poland, New Zealand, and Italy are the countries with the most newly published papers (Supplementary Figure 3).

Among al1 the publications, 1,059 institutions have published literature related to forensic TBI except for 24 papers with no records of the institutions. The institution with the most published papers was the University of Adelaide, followed by the University of Hamburg and the University of Bern. Among the top ten institutions, six were from Germany, and two were from the USA (Supplementary Table 2).

### 3.3. Top funding agencies

In our results, only 145 (13.31%) documents have been funded by 151 funding agencies. Four agencies financed more than ten papers, and three agencies supported nine, eight, and eight reports, respectively. Other agencies funded less than five papers. The top five funding agencies with the most output were Japan, China, and Serbia (Supplementary Table 3).

### 3.4. Prolific authors and their institutions

There were 2,396 authors involved in the study. Byard RW (University of Lai, Adelaide, Australia) ranked first in the number of published papers. Six authors have published more than 15 papers, and 18 have published more than ten (Supplementary Table 4).

### 3.5. Major journals

The papers were published in 38 journals, and the detailed information of the top 10 journals with the most published was shown in Supplementary Table 5. The journal with the maximum output was the American Journal of Forensic Medicine and Pathology (*n* = 208, USA). We analyzed the cooperative relationship among the journals. The American Journal of Forensic Medicine and Pathology was most closely related to Forensic Science International (Supplementary Figure 4).

### 3.6. Top 10 cited publications

Up to September 28, 2022, the 1,089 papers were cited 12,906 times, with an average of 11.85 times per paper. Each of the top 10 most cited papers was cited 154.9 times on average. Among them, seven papers were cited more than 100 times. The most cited article was published in the Journal of Forensic Sciences with a citation of 470 (Supplementary Table 6), which topic is “Virtopsy”. Three documents discussed the application of virtual autopsy. These studies show that MRI virtopsy may offer a viable alternative to the traditional autopsy. The radiological methods of MSCT and MRI can potentially become a routine “virtual autopsy” tool in the future (12, 13). In addition, the TBI of children and infants also attracted much attention.

### 3.7. Top keywords

We extracted 3,519 keywords and selected 128 that appeared more than ten times for the visual network map (Supplementary Figure 5) and density map (Supplementary Figure 6) analysis. The 128 keywords were divided into four clusters. We also counted the top 10 occurrences in the four clusters (Supplementary Table 7). In the first cluster, “injury,” “forensic science,” and “autopsy” are the most common keywords. In the second cluster, “head,” “brain,” and “gunshot wounds” are most common, among which gunshot wounds are more studied at present. In the third cluster, the focus is on the injury of infants and children, especially “shaken baby syndrome” and “child abuse”. The fourth cluster is mainly related to laboratory research; DAI and TBI markers are the research's focus.

**Figure 4:**
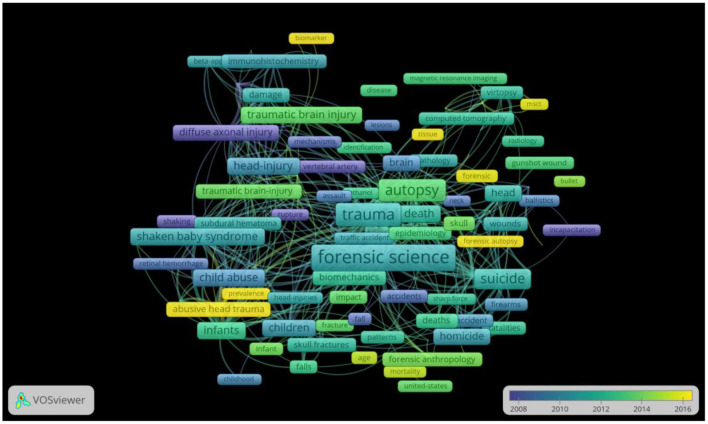
Network Visualization map for keywords plotted with VoSviewer 1.6.14. The size of the circles indicates the number of publications. The link strength between the circles reflects the frequency of co-occurrence. The different colors represented the average year the keywords appeared. The analysis method was linlog/modularity.

To inform how the field's focus has changed over time, we presented a Network Visualization map of keywords by analyzing them with the year. It showed the research topics in recent years, such as abusive head trauma, biomarker, and MSCT (Figure 4). We also made a three-field plot which showed the relationship between the keywords (research contents = left field), references authors use (intellectual roots = right field), and the top authors (center field) (Supplementary Figure 7).

## 4. Discussion

In this study, we analyzed the literature related to TBI in forensic medicine, providing the research status of TBI and its evolution over time. The output was stable before 1991 and increased with fluctuations from 1992 to 2021. The increase in publications demonstrates that TBI has attracted more and more attention in forensic science, which may be partly due to the rise in vehicles and traffic accidents globally because road traffic is the leading cause of TBI (10, 14).

North American, European, and East Asian countries have contributed much, and communications between them are more frequent than in other countries. This may be due to the systematic development of forensic medicine and comparatively large research budgets in these nations. In comparison, the voice from South America, Africa, and most of Asia is relatively low, making the knowledge of forensic TBI in these areas very limited. Given the large population in these continents, more influential studies are expected to benefit the field. The top-cited publications originating from the USA accounted for 40% of the total (15, 16). A plausible explanation is that USA researchers usually have more opportunities to attend conferences, participate in academic exchange programs, and access databases, which help to promote scholarly communication and contribute to high citation rates (17).

The term DAI appeared many times in our model, suggesting that DAI is of critical value in forensic research. Generally speaking, DAI is a brain injury mainly characterized by diffuse axonal damage to the white matter caused by head trauma, and disturbance of consciousness is a typical clinical manifestation (18). In current evidence, DAI is thought to be one of the fundamental causes of post-traumatic loss of consciousness in the absence of detectable intracranial lesions on computed tomography (CT) (19). In traffic accidents, the accurate diagnosis of DAI is of great significance for identifying death causes (10, 14). Pathological features of DAI are axonal swelling in the early post-injury period (hours to days), often without apparent hemorrhage and contusion or only with multiple punctate hemorrhages in the white matter. In this process, the shear force is often emphasized, which is usually described as seen in acceleration/deceleration trauma. When TBI occurs, the brain is subjected to multiple forces such as rotational, tensile, and compressive stress; the brain's inertia causes a separation between its relative motion and the cervical column. Thus, when the head produces rapid movement during trauma without significant physical shock, translational, rotational, and/or angular accelerations can lead to vascular stretch or axonal damage, primarily in the presence of rotational accelerations. In this case, the injury affects the plane between tissues of different densities (gray-white matter junction) and the rotational centroid of the intracranial space (rostral brainstem) (20).

Identifying such axonal damage in forensic practice is more complicated. Previously, the identification of axonal injury often depended on the detection of axonal retraction balls (21). Because all changes in the formation of axonal retraction balls occurred within about 12–24 h, axonal retraction balls are more suitable for application in delayed death cases of DAI (22). Recently, the immunohistochemical technique of β-amyloid precursor protein (β-APP) antibody has improved DAI evaluation (23–26). Under normal circumstances, β-APP will be transmitted through neurons rapidly. But axon injury will accumulate at axons' proximal and distal ends (27). Unlike traditional methods, the application of β-APP antibodies can show axonal injury even if the survival time is short. Axonal injury can be detected by β-APP within 35 min after severe TBI (28). The neuropathologist involved in forensic work is not uncommonly confronted with a case in which there is no or only a little history or, if available, the information is uncertain or is often conflicting (29, 30). Therefore, β-APP would be a valuable early marker of axonal injury in forensic neuropathology (31).

In our study, three publications regarding children and infants were involved in ten top-cited papers. TBI in children is comparable to those in adults but differs in pathophysiology. The differences are possibly ascribed to the mechanism of injuries grounded on the physical ability of the minors, age-relevant structural change, and the obstacle in the neurological evaluation of these populations (32). Even a minor injury can have severe consequences for children and infants. According to the U.S. Centers for Disease Prevention and Control data, the death rate is higher for children younger than 4 years than those of 5–14 years regarding pediatric TBI (33), which may reflect a more significant number of abusive injuries in infants and young children. In our study, the shaken baby syndrome was mentioned frequently. Shaken baby syndrome, characterized by DAI, subdural hemorrhage, and retinal hemorrhage (34), is a TBI caused by a sudden impingement or violent shaking in infants or children younger than 5 years (35). It is the leading cause of fatal head injuries worldwide in children under 2 years. For infants, controversies often exist regarding whether death after shear stress is DAI (22, 36). It is more challenging to identify shaken baby syndrome because of the particularity and difficulty in describing the condition of the infants (37). Ding et al. suggested that, when dealing with suspected shaking baby syndrome cases, the possibility of pathological temporal lobe hemorrhage should be ruled out besides careful examination of the cervical cord (34). Vázquez et al. proposed that computed tomography and magnetic resonance imaging could be auxiliary means (38). In a recent study, MRI assessment of all spinal levels and spinal cord blood sampling was recommended in cases of suspected child abuse at postmortem examination (39). On the whole, in forensic pathology, identifying traumatic brain injuries in infants and children is difficult. Further investigation is required regarding identifying this type of injury.

In clinical forensic practice, the identification of mild TBI often provokes disputes. Mild TBI may lead to severe consequences, even death in some cases. According to the American Congress of Rehabilitation Medicine's Mild TBI Committee, mild TBI is defined as a traumatically induced physiological disruption of brain function (40). The terms concussion and mild TBI are often conflated or confused. A concussion is a complex pathophysiological process affecting the brain (41). There is a lack of standards and criteria for diagnosing, without abnormal morphological changes visible to the naked eye and no positive signs in the neurological examination, which brings significant challenges to identifying concussion injuries regarding determining the level of responsibility of the parties. More objective evidence and representative indicators are needed to confirm the damage and distinguish the degree of concussion. A recent study shows that prolonged recovery of consciousness and balance impairments are associated with elevated glial fibrillary acidic protein (GFAP) and neuroinflammation in cortical tissue (42). In addition, salivary miRNA levels can identify the duration and characteristics of concussion symptoms (43). Considering that concussion is a clinical diagnosis that relies on subjective reporting, future studies may evaluate the characterization of miRNAs alongside functional measurements and neuroimaging for accurate diagnosis.

Some limitations should be acknowledged in our study. First, we used the WoS database instead of Scopus or Pubmed Center for analysis. Some relevant publications have been missed because the WOS database lists mostly English-language journals and articles, and non-English papers are not included (44). Next, we employed a comprehensive list of names of craniocerebral injuries; however, there may be a slight chance that some keywords have been omitted. Third, older documents are more likely to be cited frequently than recent ones (45). Forth, we have excluded basic science studies by limiting assessments to publications tagged as being involved with “Medicine Legal”, so some relevant publications have been missed. These all introduce bias in judging the academic influence of the literature. Hence, there may be discrepancies between our results and the overall publication.

## 5. Conclusion

For the first time, we analyzed the literature on TBI related to forensic science in the last five decades. Studies in this field have become extensive and more global after 1991. The United States, Germany, and Japan are the leading countries with the most participation and contribution in forensic research of TBI. The topic of DAI and children- and infants-related TBI received considerable attention. Overall, our study offered a historical overview of forensic TBI over the past decades. We hope that the changing trends in the field could provide investigators with vital topics and facilitate collaboration between research groups with complementary scientific interests regarding TBI.

## Data availability statement

The raw data supporting the conclusions of this article will be made available by the authors, without undue reservation.

## Author contributions

YW and JY wrote the manuscript. QC, XD, WL, XZ, HY, XY, and SN completed the figures and provided advice. All authors reviewed the manuscript. All authors contributed to the article and approved the submitted version.
