# Supplementary material for: A bibliometric analysis on traumatic brain injury in forensic medicine of a half-century (1972–2021)
Source: Front Neurol. 2023 Feb 2;14:913855. doi: 10.3389/fneur.2023.913855 (PMC9932540; doi:10.3389/fneur.2023.913855)

**Supplementary Figure 1.** The type of publications.


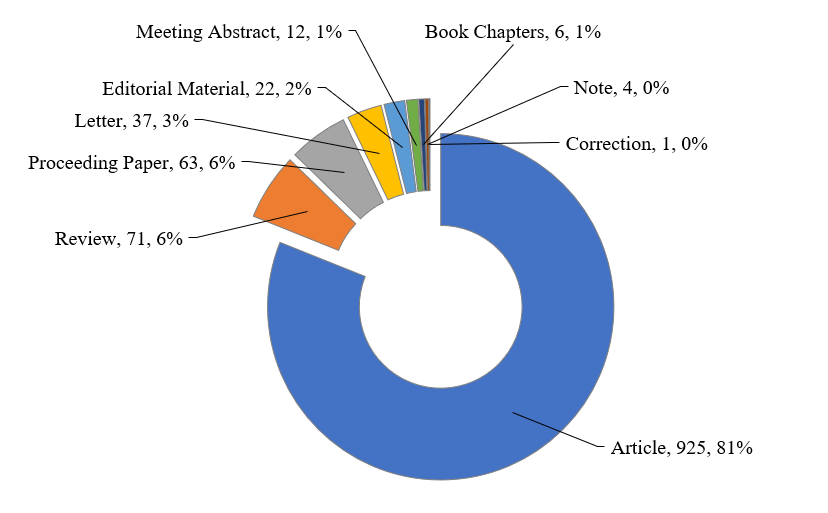


**Supplementary Figure.2.** Top 10 countries with most papers in field of craniocerebral injury.


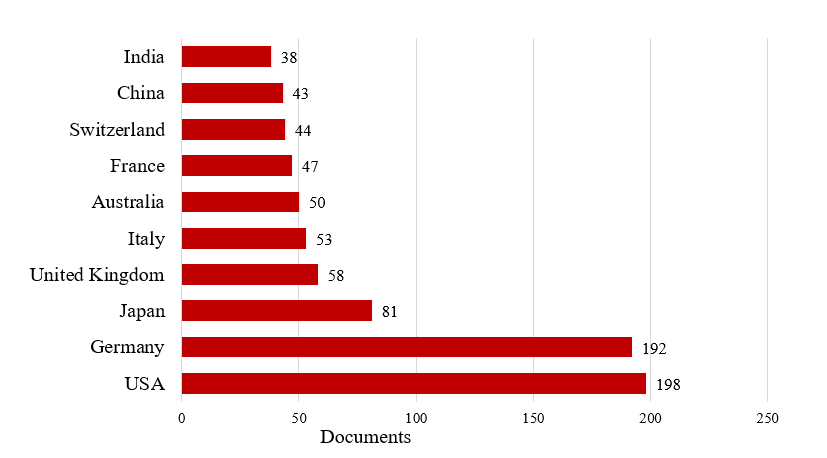


**Supplementary Figure.3.** Countries and regions co-authors relations overlay graph plotted with VOSviewer1.6.14. The size of the circles indicates the number of publications. The link strength between the circles reflects the frequency of co-occurrence. The analysis method was linlog/modularity. Scores were the average published per year.


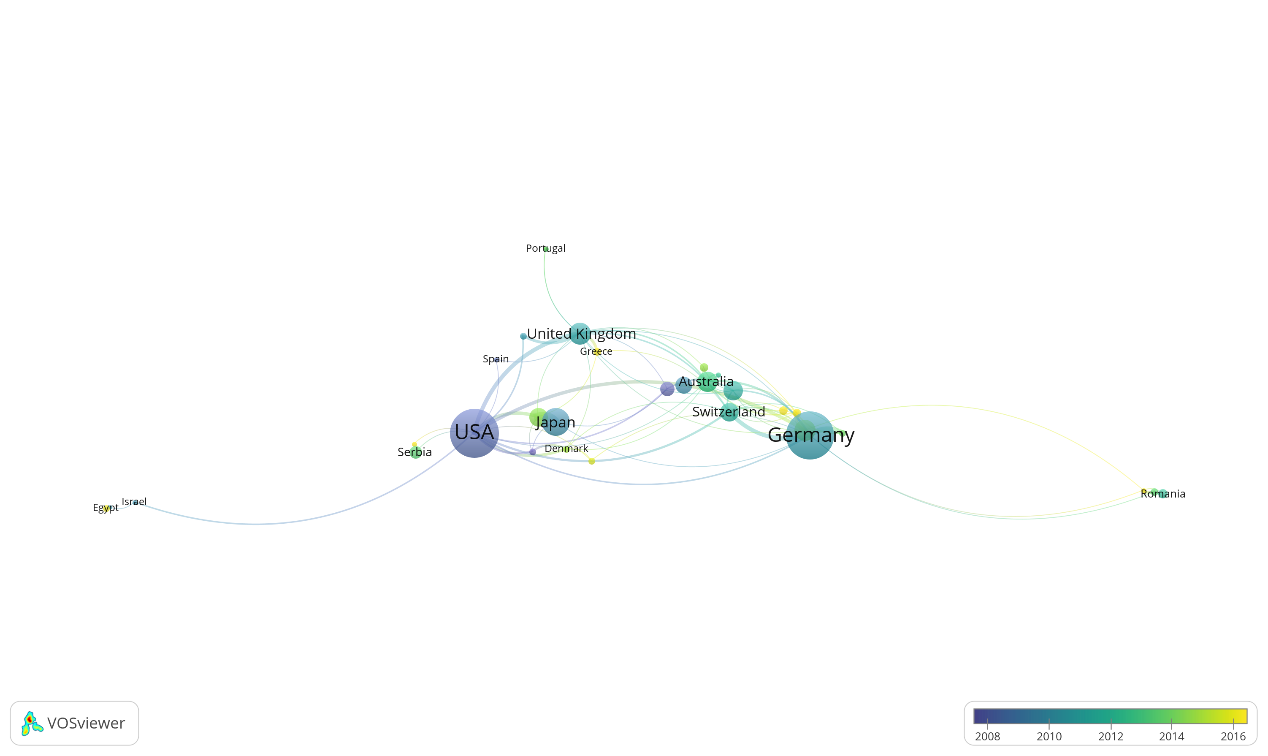


**Supplementary Figure.4.** Collaboration relations among the journals plotted with VOSviewer 1.6.14. The size of the circles indicates the number of publications. The link strength between the circles reflects the frequency of co-occurrence. The analysis method was linlog/modularity.


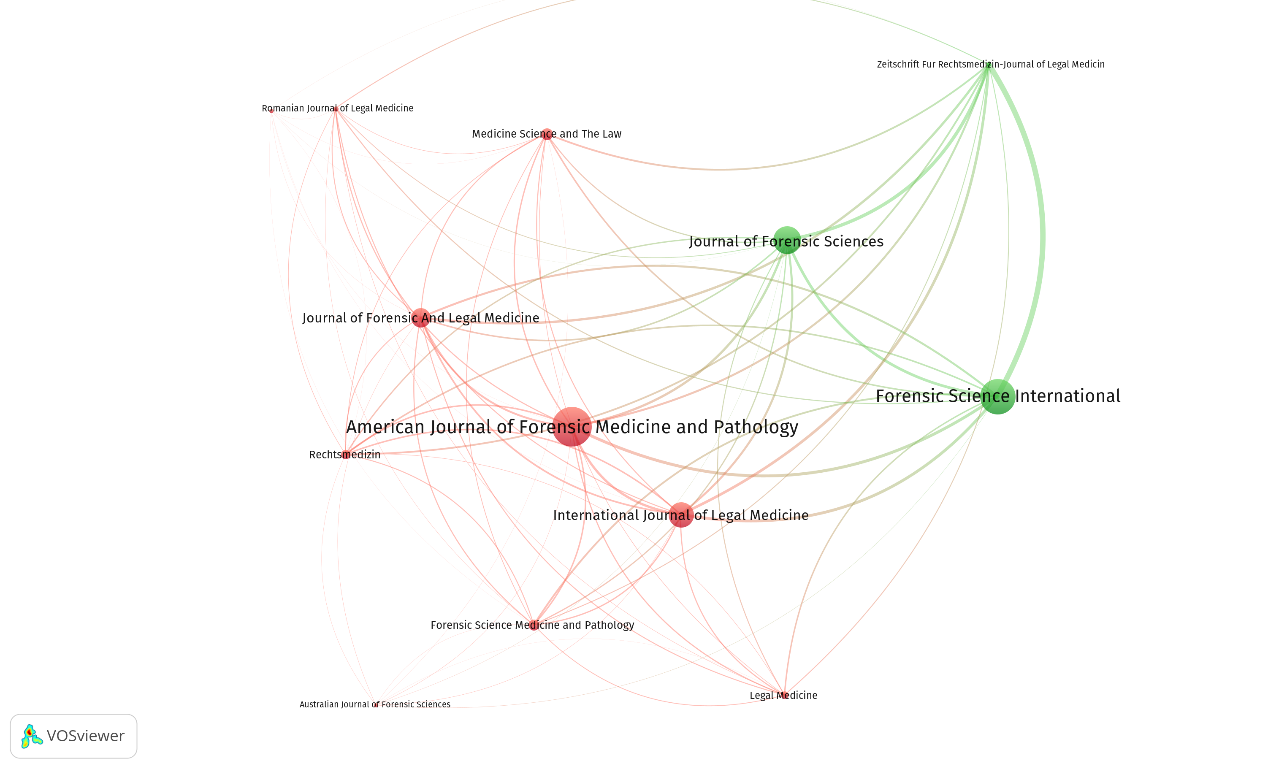


**Supplementary Figure.5.** Network Visualization map for keywords plotted with VoSviewer 1.6.14. The size of the circles indicates the number of publications. The link strength between the circles reflects the frequency of co-occurrence, each color represents a cluster of word. The analysis method was linlog/modularity.


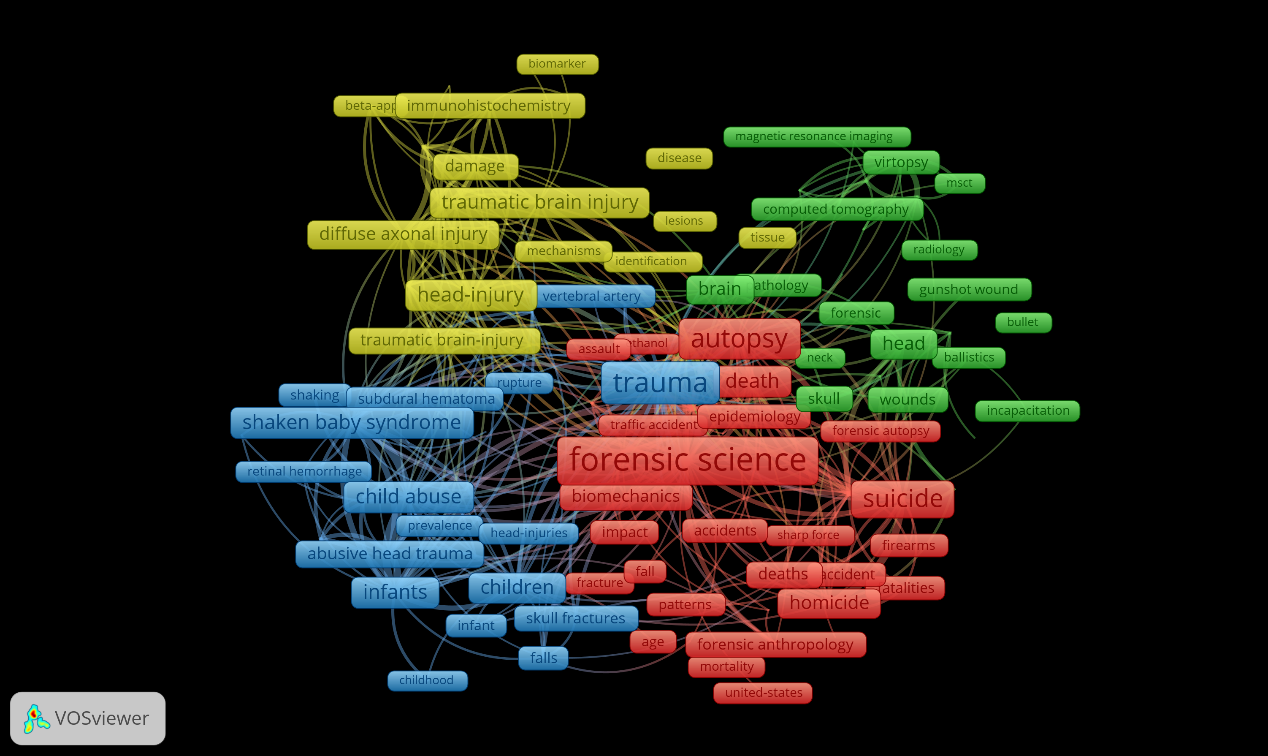


**Supplementary Figure.6.** Density visualization map for keywords plotted with VoSviewer 1.6.14. The analysis method was linlog/modularity.


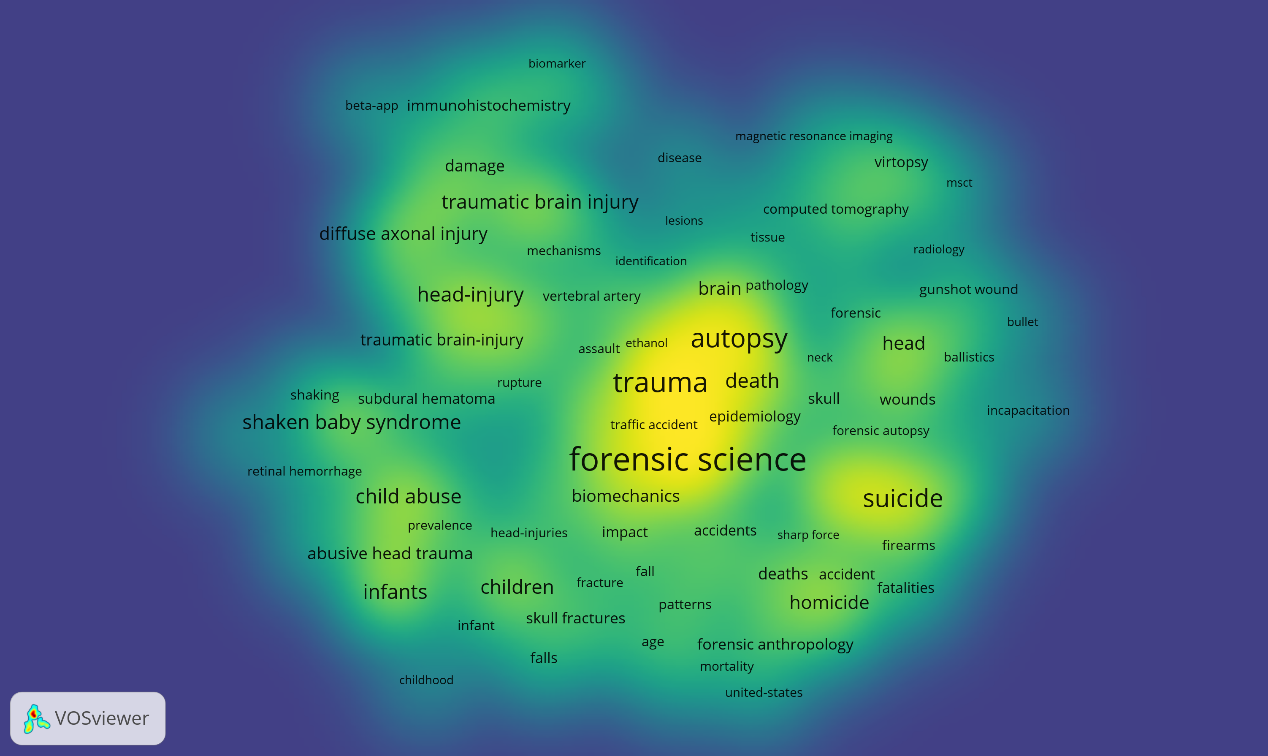


**Supplementary Figure.7.** Three Field Plot represented the cross-analysis between the keywords references authors use, and the top authors.


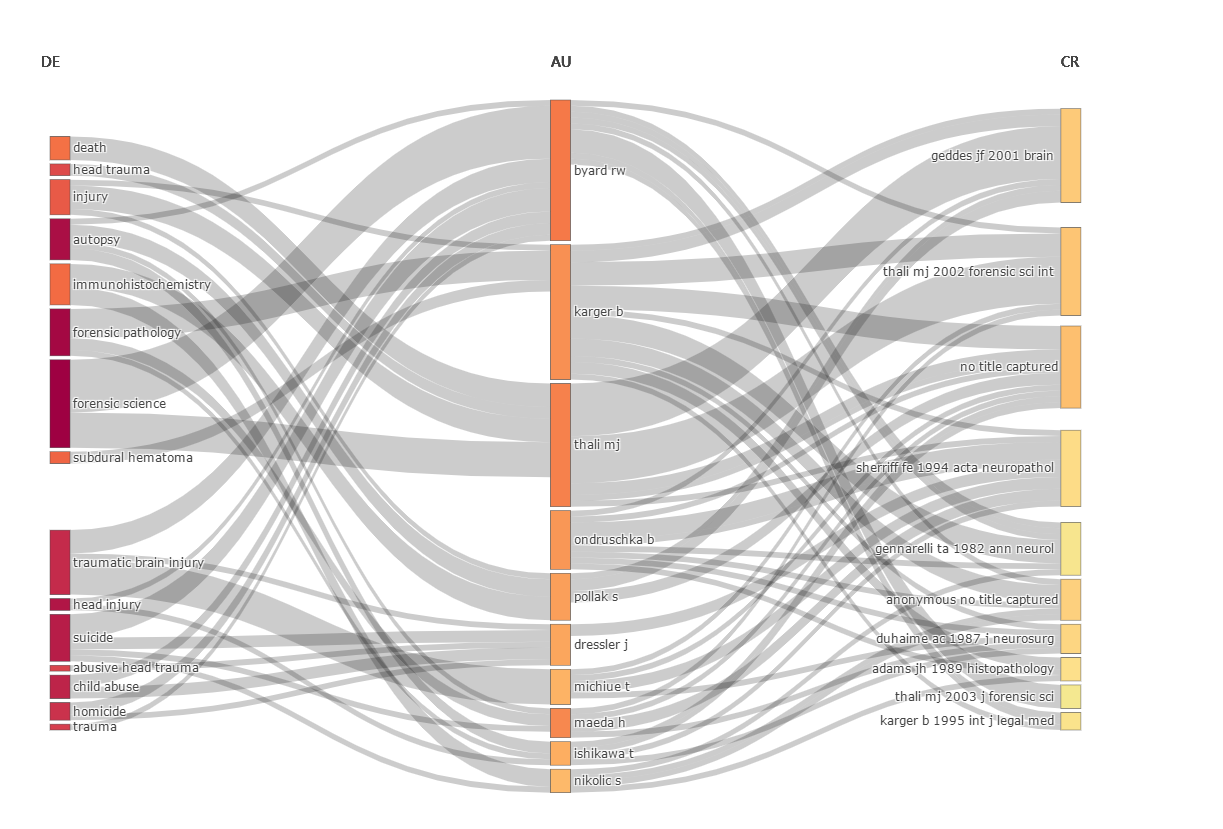

Supplement: Supplementary file 1 [file Table_1.DOCX]
