# Supplementary material for: A bibliometric analysis on traumatic brain injury in forensic medicine of a half-century (1972–2021)
Source: Front Neurol. 2023 Feb 2;14:913855. doi: 10.3389/fneur.2023.913855 (PMC9932540; doi:10.3389/fneur.2023.913855)
Supplement: Supplementary file 2 [file Table_2.DOCX]

**Supplementary Table. 1** Annual publications from 1972 to 2021.

| Years | Number of publications | Cumulative Total | AGR | RGR | Articles | Proportion of Articles |
| --- | --- | --- | --- | --- | --- | --- |
| 1972 | 1 | 1 | - | - | 0 | 0.00% |
| 1973 | 3 | 4 | 300.00% | 46.21% | 3 | 100.00% |
| 1974 | 7 | 11 | 175.00% | 14.45% | 7 | 100.00% |
| 1975 | 0 | 11 | 0.00% | 0.00% | 0 | 0.00% |
| 1976 | 3 | 14 | 27.27% | 8.04% | 3 | 100.00% |
| 1977 | 3 | 17 | 21.43% | 6.47% | 3 | 100.00% |
| 1978 | 4 | 21 | 23.53% | 5.28% | 4 | 100.00% |
| 1979 | 2 | 23 | 9.52% | 4.55% | 2 | 100.00% |
| 1980 | 3 | 26 | 13.04% | 4.09% | 1 | 33.33% |
| 1981 | 2 | 28 | 7.69% | 3.71% | 1 | 50.00% |
| 1982 | 3 | 31 | 10.71% | 3.39% | 3 | 100.00% |
| 1983 | 1 | 32 | 3.23% | 3.17% | 1 | 100.00% |
| 1984 | 2 | 34 | 6.25% | 3.03% | 2 | 100.00% |
| 1985 | 6 | 40 | 17.65% | 2.71% | 5 | 83.33% |
| 1986 | 2 | 42 | 5.00% | 2.44% | 2 | 100.00% |
| 1987 | 3 | 45 | 7.14% | 2.30% | 2 | 66.67% |
| 1988 | 0 | 45 | 0.00% | 0.00% | 0 | 0.00% |
| 1989 | 2 | 47 | 4.44% | 2.17% | 2 | 100.00% |
| 1990 | 3 | 50 | 6.38% | 2.06% | 3 | 100.00% |
| 1991 | 6 | 56 | 12.00% | 1.89% | 6 | 100.00% |
| 1992 | 17 | 73 | 30.36% | 1.56% | 14 | 82.35% |
| 1993 | 14 | 87 | 19.18% | 1.25% | 8 | 57.14% |
| 1994 | 7 | 94 | 8.05% | 1.11% | 8 | 114.29% |
| 1995 | 14 | 108 | 14.89% | 0.99% | 9 | 64.29% |
| 1996 | 13 | 121 | 12.04% | 0.87% | 13 | 100.00% |
| 1997 | 25 | 146 | 20.66% | 0.75% | 13 | 52.00% |
| 1998 | 21 | 167 | 14.38% | 0.64% | 18 | 85.71% |
| 1999 | 21 | 188 | 12.57% | 0.56% | 20 | 95.24% |
| 2000 | 13 | 201 | 6.91% | 0.51% | 7 | 53.85% |
| 2001 | 30 | 231 | 14.93% | 0.46% | 22 | 73.33% |
| 2002 | 27 | 258 | 11.69% | 0.41% | 15 | 55.56% |
| 2003 | 24 | 282 | 9.30% | 0.37% | 11 | 45.83% |
| 2004 | 22 | 304 | 7.80% | 0.34% | 16 | 72.73% |
| 2005 | 15 | 319 | 4.93% | 0.32% | 12 | 80.00% |
| 2006 | 24 | 343 | 7.52% | 0.30% | 19 | 79.17% |
| 2007 | 20 | 363 | 5.83% | 0.28% | 17 | 85.00% |
| 2008 | 39 | 402 | 10.74% | 0.26% | 34 | 87.18% |
| 2009 | 42 | 444 | 10.45% | 0.24% | 30 | 71.43% |
| 2010 | 45 | 489 | 10.14% | 0.21% | 38 | 84.44% |
| 2011 | 53 | 542 | 10.84% | 0.19% | 42 | 79.25% |
| 2012 | 61 | 603 | 11.25% | 0.17% | 50 | 81.97% |
| 2013 | 53 | 656 | 8.79% | 0.16% | 47 | 88.68% |
| 2014 | 56 | 712 | 8.54% | 0.15% | 49 | 87.50% |
| 2015 | 68 | 780 | 9.55% | 0.13% | 58 | 85.29% |
| 2016 | 47 | 827 | 6.03% | 0.12% | 40 | 85.11% |
| 2017 | 44 | 871 | 5.32% | 0.12% | 37 | 84.09% |
| 2018 | 52 | 923 | 5.97% | 0.11% | 46 | 88.46% |
| 2019 | 63 | 986 | 6.83% | 0.10% | 45 | 71.43% |
| 2020 | 55 | 1041 | 5.58% | 0.10% | 38 | 69.09% |
| 2021 | 48 | 1089 | 4.61% | 0.09% | 43 | 89.58% |

**Note:** -, no data.

**Supplementary Table. 2** Top10 organizations with most papers in field of craniocerebral injury.

| No. | Organizations | Country | Papers | Percentage % |
| --- | --- | --- | --- | --- |
| 1 | University of Adelaide | Australia | 33 | 3.03 |
| 2 | University of Hamburg | Germany | 30 | 2.76 |
| 3 | University of Bern | Switzerland | 27 | 2.48 |
| 4 | University of Munich | Germany | 25 | 2.30 |
| 5 | Udice French Research Universities | France | 22 | 2.02 |
|  | University Medical Center Hamburg Eppendorf | Germany | 22 |  |
| 6 | Free University of Berlin | Germany | 19 | 1.75 |
|  | University of Munster | Germany | 19 |  |
| 7 | University of Freiburg | Germany | 18 | 1.65 |
| 8 | Osaka Metropolitan University | Japan | 17 | 1.56 |
|  | University of Belgrade | [serbia](javascript:;) | 17 |  |
| 9 | Off Chief Med Examiner | USA | 15 | 1.38 |
|  | University of London | UK | 15 |  |
|  | University of Texas System | USA | 15 |  |
| 10 | Centre National De La Recherche Scientifique Cnrs | France | 14 | 1.29 |

**Supplementary Table. 3** Top5 funding agencies with most papers in field of craniocerebral injury.

| No. | Funding Agency | Country | Papers | Percentage % |
| --- | --- | --- | --- | --- |
| 1 | Ministry of Education Culture Sports Science and Technology Japan Mext | 24 | Japan | 2.20 |
| 2 | Japan Society for The Promotion of Science | 20 | Japan | 1.84 |
| 3 | Ministry Of Education Science Technological Development Serbia | 13 | [serbia](javascript:;) | 1.19 |
| 4 | National Natural Science Foundation of China | 10 | China | 0.92 |
| 5 | Grants In Aid for Scientific Research Kakenhi | 9 | Japan | 0.83 |

**Supplementary Table. 4** The authors’ information with the Top 5 number of papers.

| No. | Author | Papers | Percentage % | Organization | Country |
| --- | --- | --- | --- | --- | --- |
| 1 | Byard RW | 31 | 2.85 | University of Adelaide | Australia |
| 2 | Thali MJ | 25 | 2.30 | University of Zurich | Switzerland |
| 3 | Maeda H | 17 | 1.56 | Osaka City University Medical School | Japan |
| 4 | Karger B | 16 | 1.47 | Munster university | Germany |
|  | Ondruschka B | 16 | 1.47 | Univ Med Ctr Hamburg Eppendorf | Germany |
| 5 | Pollak S | 15 | 1.38 | University of Freiburg | Germany |

**Supplementary Table.5** Details of Top10 Journals with most papers.

| No. | Journal | Number | Percentage | 2021 IF | 5 years IF | 2021quartile |  |
| --- | --- | --- | --- | --- | --- | --- | --- |
|  |  |  |  |  |  |  |  |
| 1 | American Journal of Forensic Medicine and Pathology | 208 | 19.10 | 1.108 | 0.939 | Q4 |  |
| 2 | Forensic Science International | 186 | 17.08 | 2.676 | 2.662 | Q2 |  |
| 3 | Journal of Forensic Sciences | 144 | 13.22 | 1.717 | 1.654 | Q3 |  |
| 4 | International Journal of Legal Medicine | 133 | 12.21 | 2.791 | 2.602 | Q1 |  |
| 5 | Journal of Forensic and Legal Medicine | 102 | 9.37 | 1.691 | 1.786 | Q3 |  |
| 6 | Medicine Science and The Law | 63 | 5.79 | 2.051 | 1.485 | Q2 |  |
| 7 | Forensic Science Medicine and Pathology | 58 | 5.33 | 2.456 | 2.382 | Q2 |  |
| 8 | Rechtsmedizin | 49 | 4.50 | 1.112 | 0.678 | Q4 |  |
| 9 | Legal Medicine | 40 | 3.67 | 2.017 | 1.746 | Q4 |  |
| 10 | Romanian Journal of Legal Medicine | 27 | 2.48 | 0.459 | 0.398 | Q4 |  |

**Supplementary Table. 6** The information of top10 cited papers

| No. | Title | Measures | Sample size | Results/outcomes | IF*2021/ 2021 quartile | Years | Country | Citations |  |
| --- | --- | --- | --- | --- | --- | --- | --- | --- | --- |
|  |  |  |  |  |  |  |  |  |  |
| 1 | Virtopsy, a new imaging horizon in forensic pathology: Virtual autopsy by postmortem multislice computed tomography (MSCT) and magnetic resonance imaging (MRI) - a feasibility study | MSCT and MRI (Virtopsy) | 40 | Radiology was superior to autopsy in revealing certain cases of cranial, skeletal, or tissue trauma. Some forensic vital reactions were diagnosed equally well or better using MSCT/MRI. | 1.717/Q3 | 2003 | Switzerland | 470 |  |
| 2 | Long-term intracerebral inflammatory response after traumatic brain injury | - | - | Microglial activity elevated in long-term survivors of head injury and the suggestion of an association between the extent of this activity and interleukin-1 genotype. | 2.676/Q2 | 2004 | UK | 178 |  |
| 3 | Chronic Traumatic Encephalopathy, Suicides and Parasuicides in Professional American Athletes The Role of the Forensic Pathologist | Forensic autopsies and immunohistochemical analyses | 5 | CTE can only be definitively diagnosed by direct tissue examination. Forensic pathologists will play a vital and central role in the emerging disease surveillance of CTE | 1.108/Q4 | 2010 | USA | 150 |  |
| 4 | Image-guided virtual autopsy findings of gunshot victims performed with multi-slice computed tomography (MSCT) and magnetic resonance imaging (MRI) and subsequent correlation between radiology and autopsy findings | MSCT and MRI (Virtopsy) | 8 | With the spiral CT and MRI examinations and the subsequent 2D multi-planar reformation (MPR) and 3D shaded surface display (SSD) reconstruction, the entire gunshot-created complex skull fractures and brain injuries (such as wound channels and deeply-driven bone splinters) could be documented in complete and graphic detail.the radiological methods of MSCT and MRI have the potential to become a routine “virtual autopsy” tool in the future. | 2.676/Q2 | 2003 | Switzerland | 132 |  |
| 5 | Position paper on fatal abusive head injuries in infants and young children | - | - | - | 1.108/Q4 | 2001 | USA | 127 |  |
| 6 | Fatal pediatric head injuries caused by short-distance falls | - | 18 | An infant or child may suffer a fatal head injury from a fall of less than 3 meters (10 feet). The injury may be associated with a lucid interval and bilateral retinal hemorrhage. | 1.108/Q4 | 2001 | USA | 120 |  |
| 7 | Post-mortem forensic neuroimaging: Correlation of MSCT and MRI findings with autopsy results | MSCT and MRI (Virtopsy) | 57 | CT in particular possessed certain advantages in comparison with autopsy with regard to forensic reconstruction. MRI showed forensically relevant findings not seen during autopsy in several cases. | 2.676/Q2 | 2007 | Austria | 101 |  |
| 8 | Autopsy features relevant for discrimination between suicidal and homicidal gunshot injuries | - | 624 | Some bullet path directions cannot be considered indicative of suicide: downwards and back-to-front in gunshots to the temple, left-to-right in gunshots to the left chest and downwards in mouth shots. The isolated autopsy findings can only be indicative of suicide or homicide. | 2.791/Q1 | 2002 | Germany | 97 |  |
| 9 | Systemic and ocular findings in 169 prospectively studied child deaths-retinal hemorrhages usually mean CHILD-ABUS | - | 169 | In the absence of a verifiable history of a severe head injury or life-threatening central nervous system disease, retinal and ocular hemorrhages were diagnostic of child abuse. | 2.676/Q2 | 1994 | USA | 90 |  |
| 10 | The Skin-skull-brain model: a new instrument for the study of gunshot effects | - | - | "Skin-skull-brain model" has some significant advantages: the model is inexpensive, easy to construct, instantly available for use, and eliminates ethics conflicts. | 2.676/Q2 | 2002 | Switzerland | 84 |  |

Note: -, no data.

**Supplementary Table.7** Top 10 keywords in per cluster.

| No. | Cluster1 | Occurrences | No. | Cluster2 | Occurrences |
| --- | --- | --- | --- | --- | --- |
| 1 | injury | 227 | 1 | head | 55 |
| 2 | forensic science | 157 | 2 | brain | 50 |
| 3 | autopsy | 108 | 3 | gunshot wounds | 35 |
| 4 | suicide | 94 | 4 | skull | 32 |
| 5 | head injury | 75 | 5 | wounds | 29 |
| 6 | death | 98 | 6 | reconstruction | 27 |
| 7 | homicide | 56 | 7 | virtopsy | 26 |
| 8 | head trauma | 50 | 8 | gunshot | 25 |
| 9 | biomechanics | 42 | 9 | forensic radiology | 23 |
| 10 | forensic anthropology | 28 | 10 | computed tomography | 21 |
| No. | Cluster3 | Occurrences | No. | Cluster4 | Occurrences |
| 1 | trauma | 126 | 1 | forensic pathology | 105 |
| 2 | child abuse | 66 | 2 | head-injury | 63 |
| 3 | infants | 64 | 3 | traumatic brain injury | 58 |
| 4 | shaken baby syndrome | 63 | 4 | diffuse axonal injury | 50 |
| 5 | children | 58 | 5 | diagnosis | 46 |
| 6 | abusive head trauma | 43 | 6 | traumatic brain-injury | 35 |
| 7 | brain-injury | 39 | 7 | damage | 34 |
| 8 | hemorrhage | 32 | 8 | neuropathology | 33 |
| 9 | subarachnoid hemorrhage | 30 | 9 | immunohistochemistry | 30 |
| 10 | skull fractures | 28 | 10 | axonal injury | 27 |
